# The Use of the Internal Jugular Vein Collapsibility Index (IJVCI) for the Prediction of Postspinal Hypotension in Geriatric Patients Posted for Lower Limb Surgeries

**DOI:** 10.7759/cureus.76824

**Published:** 2025-01-02

**Authors:** Kirandeep Kaur, Harpriya Sara, Geetashu Duggal, Aparna Depuru, Ashwini Reddy, Namita Bansal

**Affiliations:** 1 Anesthesiology, Maharishi Markandeshwar Institute of Medical Sciences and Research (MMIMSR), Mullana, IND; 2 Anesthesiology, Duke University Medical Center, Durham, USA; 3 Anesthesiology, Dr. B.R. Ambedkar State Institute of Medical Sciences, Mohali, IND; 4 Statistician, Private Practice, Chandigarh, IND

**Keywords:** hypotension, ijvci, psh, sab, sonography

## Abstract

Background and aims

Hypotension is a recognized consequence of subarachnoid block (SAB). Sonographic measurements of inferior vena cava (IVC) index have been explored to assess intravascular volume. Internal jugular vein (IJV) assessment as a surrogate has been used in pregnant patients, but data is lacking in geriatric patients. Hence, we performed this study to find the ability of the IJV collapsibility index (IJVCI) to predict postspinal hypotension (PSH).

Materials and methods

This prospective observational study was conducted in 60 years above American Society of Anaesthesiologists (ASA) I-III patients undergoing surgeries under SAB. Ultrasonography of the IJV was done in a supine position, and IJVCI% was calculated. The baseline hemodynamic parameters were noted, followed by every one minute for three minutes and every five minutes till 20 minutes after spinal anesthesia. Decrease in systolic blood pressure (SBP) of more than 20% from baseline or mean arterial pressure (MAP) of less than 65 mm Hg was considered as PSH. Quantitative variables and categorical data were compared using the Mann-Whitney U test and chi-square test, respectively. A p-value < 0.05 was considered statistically significant.

Results

Out of 52 patients, 29 (55.76%) developed PSH, seen commonly among ASA III patients and patients with higher baseline SBP. IJVCI was significantly higher in the hypotensive group with mean values of 53.11±11.52 versus 40.96±12.20 in the non-hypotensive group. The receiver operator characteristics curve (ROC) was drawn with an area under the curve (AUC) value of 0.769 with a sensitivity of 89.7% and specificity of 69.6%.

Conclusion

The simplicity and noninvasiveness of IJV sonography make IJVCI useful for predicting PSH in elderly patients.

## Introduction

Subarachnoid block (SAB) is the preferred technique for lower limb surgeries in the geriatric population. SAB is advantageous over general anesthesia for its simplicity of procedure, avoiding polypharmacy, avoiding undue airway manipulations, reducing the risk of venous thrombosis, decreasing blood loss, and minimal interference with cognition in the perioperative period [1,2].

Postspinal hypotension (PSH) is a recognized complication with an incidence of approximately 30% [3]. Intraoperative hypotension has been associated with high morbidity and mortality, including intraoperative nausea and vomiting, increased risk of acute kidney injury (AKI), perioperative myocardial infarction (MI), ischemic stroke, increased hospital stays, postoperative delirium, and cognitive dysfunction [4].

Due to the changes in physiology, PSH is exaggerated in elderly patients. SAB-induced sympatholysis in a relatively higher resting sympathetic tone and decreased baroreceptor activity of the elderly exposes them to a greater fall in blood pressure [5]. SAB causes sympathetic blockade, which decreases systemic vascular resistance, precipitating relative hypovolemia. To prevent PSH, preloading/co-loading before SAB remains empirical therapy. Also, for the treatment of PSH, the first step is primarily giving a fluid bolus. There are studies for prophylactic use of vasopressors such as phenylephrine, ephedrine, or norepinephrine to prevent PSH. However, excess fluid carries the risk of volume overload, and vasopressors may cause malignant arrhythmias, which may decompensate already aging physiology [6,7]. Thus, identifying the potential patients with latent hypovolemia who are at risk of PSH can help prevent its complications.

Noninvasive measures such as pleth variability index, near-infrared spectroscopy, and inferior vena cava collapsibility (IVCC) have been previously studied in different groups of patients. Sophisticated monitors mentioned above may not be available in all health care centers, and measuring inferior vena cava sonography may not be possible in all subsets of patients, including morbidly obese patients, patients with bowel gas, emphysematous lungs, pregnant females, and post-op cases of upper abdominal surgery. The internal jugular vein collapsibility index (IJVCI) can be used as a surrogate for IVCC. It is the one vein with which most anesthesiologists are familiar, easily accessible by ultrasound, and has an easier learning curve [8,9].

Most of the studies in the literature using IJVCI have been done in pregnant patients [10]. However, there is a paucity of literature aimed at evaluating the role of IJVCI in the prediction of intravascular volume status and hence the risk of PSH in the geriatric population. Thus, we conducted this prospective observational study with the aim of evaluating the role of IJVCI using ultrasonography (USG) to predict PSH in geriatric patients undergoing lower limb surgeries. 

## Materials and methods

**Figure 1:**
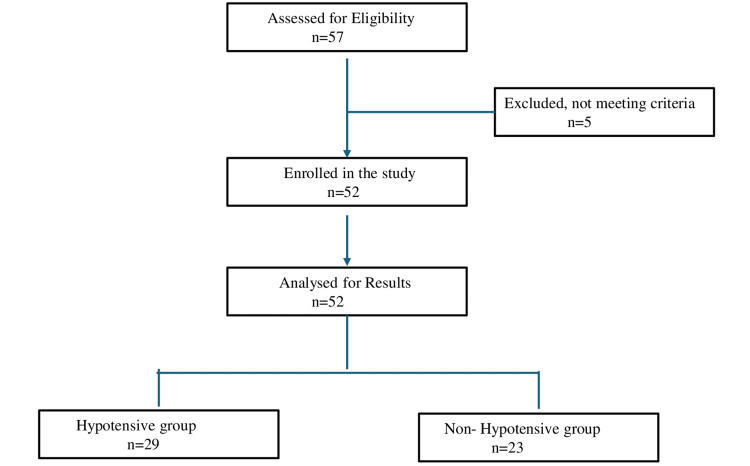
Flow diagram

After getting approval from the institutional ethics committee (Project no. 3003), we registered this single-cohort prospective observational study at Clinical Trials Registry-India (CTRI) (CTRI/2024/09/073847). The study was carried out over a period of three months (September 14, 2024 to December 15, 2024) in accordance with the Helsinki Declaration after the ethics committee’s approval. We included a total of 52 American Society of Anaesthesiologists (ASA) I-III patients of either gender aged 60 years or above having a body mass index (BMI) of 20-30 kg/m^2^ posted for elective lower limb surgeries under spinal anesthesia. Pregnant patients, those with a history of severe cardiovascular, renal disease, and hepatic disorders; the obese (BMI>30kg/m^2^) and emergency cases were excluded (Figure 1). This study was done following the strengthening the reporting of observational studies in epidemiology (STROBE) guidelines.

After obtaining written informed consent and confirming nil per oral status as per ASA guidelines, the patient was shifted to the operating room, baseline monitors were attached, and baseline vitals were noted. The patient was placed in a supine position, and a linear ultrasound probe (7-13 Hz) was placed horizontally 2 cm superior to the right sternoclavicular joint with minimal pressure to maintain the round contour of the IJV. The sonographic measurements were assessed using M-mode imaging. The maximum and minimum diameters (mm) of the IJV were measured during maximum inspiration and expiration.

The IJVCI was calculated as [(maximum diameter - minimum diameter) / maximum diameter] x 100.9.

The anesthesiologist performing preoperative ultrasonography and the baseline parameter recording were not involved in the intraoperative monitoring and management. The baseline values of heart rate (HR), systolic blood pressure (SBP), diastolic blood pressure (DBP), and mean arterial pressure (MAP) were recorded. Co-loading with Ringer’s lactate at 7 ml/kg was done using an 18 G IV cannula. Under strict aseptic precautions, SAB was performed in the sitting position in L3-4 or L4-5 spaces with a 25-gauge Quincke’s needle, and 2.6 ml of 0.5% bupivacaine heavy was administered at a rate of 0.2 ml per second after verifying constant flow of cerebrospinal fluid. A sensory level of T8 was targeted and assessed using a cold alcohol swab. Hemodynamic parameters were noted at pre-induction (T0), then at one, two, and three minutes (T1, 2, 3) after SAB and the fifth minute onwards for every five minutes (T4, 5, 6, 7) till 20 minutes. Any decrease in SBP of more than 20% from the baseline value or MAP less than 65 mm Hg was considered PSH and was treated with Inj. mephentermine 3-6 mg IV in incremental doses.

Statistical analysis

This was a prospective observational study, and based on the primary objective, the sample size was calculated. From prior studies, it was found that about 46.8% developed hypotension, and IJVCI was found to be significantly different between the two groups (hypotensive and non-hypotensive) [8]. Hence, a sample size of 52 patients was calculated, which achieved a power of 80% and a significance level of 0.05.

Data were described in terms of range, mean ± standard deviation (± SD), frequencies (number of cases), and relative frequencies (percentages) as appropriate. Comparison of quantitative variables between the study groups was done using the Mann-Whitney U test. For comparing categorical data, the chi-square (χ2) test was performed, and the Fisher exact test was used when the expected frequency is less than 5. The receiver operator characteristics (ROC) curve was done, and the criterion value was estimated depending on the specificity and sensitivity. The area under curve (AUC) was measured. A probability value (p-value) less than 0.05 was considered statistically significant. All statistical calculations were done using IBM SPSS Statistics for Windows, Version 21 (Released 2012; IBM Corp., Armonk, New York, United States).

## Results

Fifty-two patients were enrolled and completed the study. Patient demographics and baseline data are presented in Table 1. In the current study, 29 out of 52 patients (55.76%) developed PSH. The patients were divided into two groups, hypotensive and non-hypotensive. Age, gender, and BMI were found to be comparable between the two groups, with more frequent hypotensive episodes noted among ASA III patients and patients with a higher baseline SBP (155.31±21.67 versus 142.74±11.67). However, baseline values of DBP, MAP, and HR were statistically insignificant between the two groups (Table 1). The maximum fall in SBP, DBP, and MAP was seen at five minutes after SAB, and it continued to be significant till 20 min. We did not gather data after 20 min of SAB. HR also decreased in both groups, 85.38±8.81 to 77.48±9.17 in the hypotensive group and 81.17±10.89 to 75.43±11.60 in the non-hypotensive group, but the drop in HR was not statistically significant (Table 2).

**Table 1:** Patient demographics and baseline data Data presented as n: number and mean (±) standard deviation (SD) BMI: body mass index); ASA-PS: American Society of Anaesthesiologists-physical status; HR: heart rate; SBP: systolic blood pressure; DBP: diastolic blood pressure; MAP: mean arterial pressure

Parameters	Non-hypotensive (n=23) Mean(±)SD	Hypotensive (n=29) Mean(±)SD	p-value
Age (years)	66.39(±)6.53	65.90(±)2.76	0.714
Gender n (M)	6	7	0.872
(F)	17	22	
BMI (kg/metre^2^)	23.69(±)2.70	24.07(±)3.00	0.636
ASA-PS n I	9	2	0.003
II	14	21	
III	0	6	
Baseline HR (beats/min)	81.17(±)10.89	85.38(±)8.81	0.130
Baseline SBP (mm/hg)	142.74(±)11.67	155.31(±)21.67	0.016
Baseline DBP (mm/hg)	87.96(±)11.06	85.79(±)7.17	0.398
Baseline MAP (mm/hg)	106.00(±)10.74	108.69(±)6.06	0.260

**Table 2:** Intraoperative hemodynamic parameters Data presented as mean (±) standard deviation (SD) T1, T2, T3, T4, T5, T6, and T7 are time at 1, 2, 3, 5, 10, 15, and 20 min postspinal, respectively. SBP: systolic blood pressure; DBP: diastolic blood pressure; MAP: mean arterial pressure; HR: heart rate

Parameters	Time	Non-hypotensive (n=23) Mean(±)SD	Hypotensive (n=29) Mean(±)SD	p-value
SBP	T1	138.09(±)14.03	144.28(±)32.17	0.395
	T2	132.87(±)14.13	134.62(±)24.12	0.759
	T3	128.78(±)11.83	125.14(±)21.33	0.466
	T4	126.22(±)9.16	117.14(±)17.88	0.031
	T5	126.70(±)10.42	111.93(±)18.40	0.001
	T6	126.17(±)8.99	113.52(±)12.23	0.000
	T7	127.39(±)10.68	112.55(±)13.71	0.000
DBP	T1	86.83(±)12.69	84.24(±)8.84	0.391
	T2	80.57(±)9.41	83.34(±)6.18	0.206
	T3	80.83(±)9.70	79.38(±)9.26	0.586
	T4	78.43(±)8.16	71.45(±)11.74	0.019
	T5	75.74(±)7.89	68.83(±)15.29	0.055
	T6	78.61(±)10.10	71.24(±)11.23	0.018
	T7	78.04(±)8.27	70.86(±)7.92	0.002
MAP	T1	103.35(±)11.43	103.90(±)15.41	0.887
	T2	98.74(±)9.53	99.52(±)7.73	0.746
	T3	96.43(±)9.29	94.28(±)11.50	0.469
	T4	94.09(±)7.57	86.31(±)11.41	0.007
	T5	92.61(±)7.90	82.86(±)14.47	0.006
	T6	94.22(±)9.21	85.00(±)9.70	0.001
	T7	94.26(±)8.09	84.41(±)9.05	0.001
HR	T1	80.52(±)10.84	84.34(±)11.76	0.234
	T2	80.17(±)13.42	83.28(±)11.15	0.367
	T3	82.00(±)17.01	79.52(±)12.27	0.544
	T4	80.17(±)15.07	80.10(±)12.56	0.985
	T5	77.39(±)10.96	79.52(±)10.75	0.486
	T6	75.13(±)9.13	79.41(±)10.87	0.137
	T7	75.43(±)11.60	77.48(±)9.17	0.480

IJVCI was significantly higher in the hypotensive group, with mean values of 53.11±11.52 versus 40.96±12.20 in the non-hypotensive group with a p-value of 0.001. The cut-off value in our study was 46.04%. The maximum and minimum IJV diameters were statistically insignificant. IJV maximum diameter was 11.42±3.47 mm versus 9.80±2.45 mm, and IJV minimum diameter was 5.11±1.14 versus 5.79±1.83 mm in the hypotensive and non-hypotensive groups, respectively.

**Figure 2:**
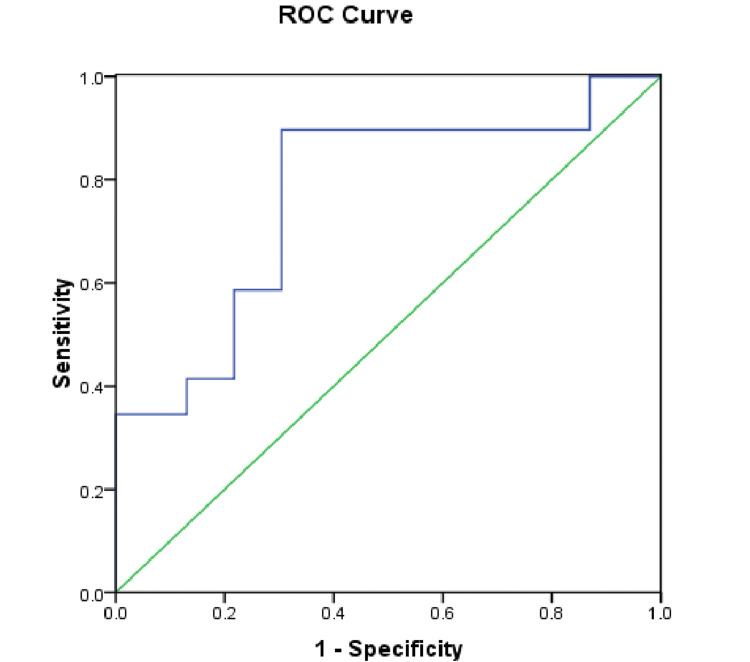
ROC curve ROC: receiver operator characteristics

The ROC curve was drawn using IJVCI to assess its ability to predict PSH (Figure 2). The curve we obtained had an area under the curve value of 0.769 with moderate predictive ability at a sensitivity of 89.7% and a specificity of 69.6%.

## Discussion

In the current study, a decrease in SBP of >20% of baseline or MAP <65 mm Hg were targets to define hypotension. We included PSH for up to 20 minutes after SAB and sensory levels up to T8. This could avoid confounding factors, including positioning, type of procedure, duration of surgery, and surgical bleed.

Preoperative volume status can be evaluated by invasive and non-invasive modalities. USG of IVC is a non-invasive and well-validated examination that is often used in emergencies and critical care settings to measure central venous pressure (CVP). However, in recent times, studies have verified that IJV sonography can be used as a surrogate for volume assessment and has a similar ability to predict PSH [9,10]. Most of the studies have been done in pregnant patients and young adults. There is scarce literature depicting IJVCI for predicting PSH in the elderly.

In our study, 55.76% of patients had PSH, more so in ASA III patients. The primary finding of the study was IJVCI to predict PSH. We found that the cut-off value of 46.04% was moderately able to predict hypotension after the SAB. The ROC curve constructed in our study had an AUC of 0.769 with a sensitivity of 89.7% and a specificity of 69.6% at 95% CI. In a study by Kilic et al., 22 patients (46.8%) developed hypotension after spinal anesthesia, and IJVCI was a reasonable predictor of PSAH. They reported an AUC curve of 0.709, with sensitivity and specificity of 64% and 63.6%, respectively, with a cut-off point of 22.6% [8]. In another study, Elbadry et al. found that a cut-off value of 38.5% for the IJVCI could predict PSH in pregnant patients [10]. Similarly, Abdelhamid et al. found the median value of 38.09% for IJVCI was an independent predictor of hypotension [11]. However, the differences in sample size, different volumes of drugs, different drugs, levels of SAB, and variable targets for hypotension hinder the generalizability of the findings of different studies. In addition, the differences in population age and physiological changes between the current work and the above studies may explain differences in their results compared to ours.

In the current study, none of the maximum and minimum diameters of IJV were found to have a statistically significant association with hypotension. This was contrary to the findings of Avcil M. et al., who have reported both minimum and maximum diameters of IJV to correlate with CVP and volume status. They demonstrated an increase in the maximum IJV of more than 1.01 cm and a decrease in the minimum IJV of less than 0.71 cm as potential predictors of a CVP value equivalent to 5 mmHg [12]. In another study by Abdelhamid et al., the authors found cut-off values of 0.91 cm and 0.59 cm, respectively, for the maximum and minimum diameters of IJV to predict hypotension [11]. The above differences in studies could be due to the variable techniques of acquiring venous dimensions, different positions of the patients, and respirophasic variations in different subsets of patients. Also, different statistical tests and operator experience can cause differences in the findings and their association with hypotension.

Baseline SBP was significantly associated with PSH in the current study. The mean baseline SBP was 155.31±21.67 mm Hg in the hypotension group versus 142.74±11.67 mm Hg in the non-hypotensive group of patients, which was statistically significant (p-value 0.016). Our results were in line with the previous studies concluding a higher fall in blood pressure in hypertensive patients [13,14]. This finding may be explained because of increased sympathetic activity due to the loss of elasticity in the arterial wall and hypertension-induced structural changes that in turn result in a decrease in blood pressure due to sympathetic blockade with SAB. Also, a statistically significant drop in SBP was seen five minutes onwards. Our results were similar to Chekol et al., where the majority of patients had maximum hypotension at five minutes and continued up to 15 min; thereafter it started to decrease in magnitude [15]. The reason for hypotensive episodes in five to 25 minutes may be because of the drug in SAB effects on sympathetic, sensory, and motor blockage.

However, baseline DBP, MAP, and HR were not statistically significant in our study. These findings were comparable to a study done on elderly patients assessed for PSH, where they found baseline HR but not MAP was significantly higher in the hypotensive group [11]. Another study on pregnant patients also found no statistically significant difference between hypotensive and non-hypotensive patients after SAB [16].

Limitations

Our study had certain limitations. We recruited physiologically preserved patients in a small sample size. Preoperative antihypertensives and other medications were not protocolized in our study. We did not compare our findings with more validated techniques such as the IVC collapsibility index. We did not follow our patients for postoperative outcomes in a hypotensive group. Future studies may be planned using dynamic parameters into consideration along with IJVCI.

## Conclusions

Due to the simplicity of IJV sonography for perioperative use and its non-invasive character, IJVCI can be used as an index with moderate predictive value for elderly patients undergoing surgery in spinal anesthesia.
